# Facilitators of and Barriers to Accessing Hospital Medical Specialty Telemedicine Consultations During the COVID-19 Pandemic: Systematic Review

**DOI:** 10.2196/44188

**Published:** 2023-07-10

**Authors:** Ana Soraia Cunha, Ana Rita Pedro, João V Cordeiro

**Affiliations:** 1 NOVA National School of Public Health Public Health Research Centre Universidade NOVA de Lisboa Lisbon Portugal; 2 NOVA National School of Public Health, Public Health Research Centre, Comprehensive Health Research Center, NOVA University Lisbon Lisbon Portugal; 3 Interdisciplinary Centre of Social Sciences, NOVA University Lisbon Lisbon Portugal

**Keywords:** health services accessibility, COVID-19, hospitals, barriers, facilitators, telemedicine

## Abstract

**Background:**

The COVID-19 pandemic accelerated the digital transition in health care, which required a rapid adaptation for stakeholders. Telemedicine has emerged as an ideal tool to ensure continuity of care by allowing remote access to specialized medical services. However, its rapid implementation has exacerbated disparities in health care access, especially for the most susceptible populations.

**Objective:**

We aimed to characterize the determinant factors (facilitators and barriers) of access to hospital medical specialty telemedicine consultations during the COVID-19 pandemic and to identify the main opportunities and challenges (technological, ethical, legal, and social) generated by the use of telemedicine in the context of the COVID-19 pandemic.

**Methods:**

We conducted a systematic review according to PRISMA (Preferred Reporting Items for Systematic Reviews and Meta-Analyses) guidelines. A total of 4 databases (Scopus, Web of Science, PubMed, and Cochrane COVID-19 Study Register) were searched for empirical studies published between January 3, 2020, and December 31, 2021, using established criteria. The protocol of this review was registered and published in PROSPERO (CRD42022302825). A methodological quality assessment was performed, and the results were integrated into a thematic synthesis. The identification of the main opportunities and challenges was done by interpreting and aggregating the thematic synthesis results.

**Results:**

Of the 106 studies identified, 9 met the inclusion criteria and the intended quality characteristics. All studies were originally from the United States. The following facilitating factors of telemedicine use were identified: health insurance coverage; prevention of SARS-CoV-2 infection; access to internet services; access to technological devices; better management of work-life balance; and savings in travel costs. We identified the following barriers to telemedicine use: lack of access to internet services; lack of access to technological devices; racial and ethnic disparities; low digital literacy; low income; age; language barriers; health insurance coverage; concerns about data privacy and confidentiality; geographic disparities; and the need for complementary diagnostic tests or the delivery of test results.

**Conclusions:**

The facilitating factors and barriers identified in this systematic review present different opportunities and challenges, including those of a technological nature (access to technological devices and internet services and level of digital literacy), a sociocultural and demographic nature (ethnic and racial disparities, geographic disparities, language barriers, and age), a socioeconomic nature (income level and health insurance coverage), and an ethical and legal nature (data privacy and confidentiality). To expand telemedicine access to hospital-based specialty medical consultations and provide high-quality care to all, including the most susceptible communities, the challenges identified must be thoroughly researched and addressed with informed and dedicated responses.

## Introduction

### Background

The COVID-19 pandemic, declared on March 11, 2020, by the World Health Organization (WHO), has drastically impacted health care delivery [1,2]. Health care professionals, patients, and other stakeholders in the health sector had to adapt to a new reality to try to contain viral spread and prevent disease [3,4]. Furthermore, governments have implemented containment measures and infection control strategies, including social distancing and isolation [3,5].

Information and communication technologies have played a fundamental role in facilitating contact between people, enabling access to work, education, and health care services during the COVID-19 pandemic [6,7]. In the health care field, as preventive measures were put in place, the number of consultations and face-to-face contacts between patients and health professionals have decreased [8,9]. Therefore, telemedicine has established itself as a definitive digital solution to address the challenges posed by COVID-19–related isolation measures [1,10-12]. The WHO defines telemedicine as “the delivery of health services, where distance is a critical factor, by all health professionals using information and communication technologies for the exchange of valid information for the diagnosis, treatment and prevention of disease and injury, research and evaluation, and for the continuing education of health care providers, all in the interest of advancing the health of individuals and their communities” [13]. Telemedicine is recognized as one of the main tools for the development of current health systems, with interventional potential in diagnosing, treating, and preventing diseases [14-16]. Originally designed to improve health care access in remote communities or across distant geographic locations, telemedicine has been gradually implemented in health care organizations over the last decade [1,10,17]. Different reasons explain telemedicine’s slow uptake, including limitations in administrative regulations, the absence of solid legal frameworks, low investment in technological resources, and the reluctance of professionals and users to adopt digital solutions [1,18,19]. The COVID-19 pandemic significantly accelerated the implementation of telemedicine [20-23]. In the last 2 years alone, telemedicine has been implemented in primary and secondary care across several medical specialties and has assisted in screening, diagnosis, consultations, follow-up visits, access to laboratory results, and medical advice [18,19,24-27].

In parallel, the most susceptible populations have been largely left out of telemedicine adoption during the COVID-19 pandemic [28]. Older patients, those of lower socioeconomic status, and those who live with chronic diseases and reside in rural or remote areas have been known to have lower access to health care [29]. Such characteristics, combined with lower literacy levels and access to technologies and internet services, have limited even more telemedicine-mediated health care access, reinforcing preexisting disparities [1,18,30-32].

Since the beginning of the pandemic, different studies have characterized the impact of telemedicine on health care access and identified related opportunities and challenges in various medical settings [26,33-42]. Considering the challenges of telemedicine adoption, particularly during the COVID-19 pandemic, it is crucial that future research focuses on identifying the facilitating factors and addressing the barriers to its widespread use. Identifying these factors and reviewing and systematizing the related evidence are essential steps toward providing a basis for future recommendations aimed at promoting the equitable adoption of telemedicine solutions.

### Objectives

This systematic review aims to characterize the determinants (facilitators and barriers) of telemedicine use regarding access to hospital medical specialty consultations and to identify the main opportunities and challenges (technological, ethical, legal, and social) generated by the use of telemedicine in the context of the COVID-19 pandemic.

## Methods

The methodology for this systematic review followed the PRISMA (Preferred Reporting Items for Systematic Reviews and Meta-Analyses) guidelines (Multimedia Appendix 1) [43]. The study protocol was registered and published in PROSPERO (CRD42022302825) [44].

### Search Strategy

Primary study collection occurred between October 15, 2021, and January 17, 2022, in PubMed, Scopus, Web of Science, and Cochrane COVID-19 Study Register databases, using the following search keys and Medical Subject Headings (MeSH) terms:

- PubMed–(“Telemedicine” [Mesh]) AND (“Health Services Accessibility” [Mesh]) AND (“COVID-19” [Mesh] OR “SARS-CoV-2” [Mesh]) AND “Healthcare Disparities” [Mesh].
- Scopus, Web of Science, and the Cochrane COVID-19 Study Register–"Telemedicine" AND "Health Services Accessibility" AND "COVID-19" OR "SARS-CoV-2" AND "Healthcare Disparities."

### Eligibility Criteria

General eligibility criteria included date of publication (from January 3, 2020, to December 31, 2021); language of the article (English, Spanish, and Portuguese); and article type (peer-reviewed original research articles, qualitative, quantitative, and mixed methods evidence published in indexed scientific journals; full-text access to the manuscript).

Inclusion and exclusion criteria were defined according to the PICOS (Population, Intervention, Comparison, Outcome, and Study Design) strategy [45], as shown in Textboxes 1 and 2.

**PICOS (Population, Intervention, Comparison, Outcome, and Study Design [45]) inclusion criteria.**

**Population (P)**

- Users of hospital medical specialty consultations via telemedicine during the COVID-19 pandemic.
- Adult and pediatric population.

**Intervention (I)**

- Studies analyzing the effect of telemedicine on access to hospital medical specialty consultations during the COVID-19 pandemic.
- Studies characterizing the potential and obstacles to accessing telemedicine services during the COVID-19 pandemic.
- Studies identifying challenges (technological, ethical, legal, and social) generated by telemedicine in accessing hospital medical specialty consultations during the COVID-19 pandemic.

**Comparison (C)**

- Not applicable

**Outcome (O)**

- Studies that analyze dimensions of hospital medical specialty consultations where telemedicine can be a key aid to improving access to care during a public health emergency.
- Studies that identify facilitators and barriers to the implementation of telemedicine in health care.
- Studies that are grounds for future recommendations related to the adoption of telemedicine in a medical specialty setting.

**Study design (S)**

- Observational studies (cohort, case-control, cross-sectional, prospective longitudinal studies).
- Descriptive studies (case studies).
- Randomized controlled trials.

**PICOS (Population, Intervention, Comparison, Outcome, and Study Design [45]) exclusion criteria.**

**Population (P)**

- Users of primary health care consultations.
- Users of addictive behavior consultations.
- Users of vaccination consultations.

**Intervention (I)**

- Studies in which telemedicine is not the main intervention.
- Studies in which telemedicine is not related to access to health care.
- Studies in which the intervention was not carried out between January 3, 2020, and December 31, 2021.

**Comparison (C)**

- Not applicable

**Outcome (O)**

- Studies that analyze how telemedicine can improve health care access exclusively outside the hospital medical specialty setting.

**Study design (S)**

- Systematic reviews, narrative reviews, or meta-analyses.
- Commentary or perspective articles.

### Selection Process and Quality Appraisal

Articles resulting from the application of the search strategy were transferred to Rayyan software (Rayyan), which was used throughout the selection process [46]. Subsequently, all duplicate articles were eliminated. Afterward, the 3 reviewers (ASC, ARP, and JVC) decided on article inclusion and exclusion based on the application of the eligibility criteria, first by analyzing the title, then by analyzing the abstract, and finally by reading the full article. In these 3 phases, selection was made independently and blindly among reviewers.

Following article selection, anonymity was lifted, and discrepancies were discussed and resolved unanimously, according to the eligibility criteria. Thereafter, selected articles were independently assessed for their quality by 2 reviewers (ARP and JVC). Considering the study designs and methodologies included in this review, quality assessment was performed using the Mixed Methods Appraisal Tool (version 2018; Canadian Intellectual Property Institute), which aims to critically analyze the methodological reliability and validity of 5 categories of studies (qualitative studies, quantitative studies [randomized and nonrandomized], quantitative descriptive studies, and mixed methods studies) [47-50]. Following individual quality assessments, discrepancies were resolved unanimously among reviewers.

### Data Extraction and Synthesis

In accordance with the Cochrane Collaboration guidelines [51], data from individual articles were extracted by the first reviewer (ASC) into a data extraction table, including the following characteristics: title and author; publication date; country of publication; period of data collection; study design; sample; setting; study objective; medical specialty identification; description of intervention; and main results.

Following data extraction, data synthesis proceeded in 2 stages. First, considering that the aim of this review is directed toward a qualitative answer, quantitative results were transformed into qualitative ones (qualitizing), as proposed by Popay et al [47,52,53]. Second, in line with the methodology proposed by Thomas and Harden [54], a thematic synthesis of the results was performed in the following sequential steps: text coding, construction of descriptive themes, and creation of analytical themes [50,54-56]. Thematic synthesis was performed and double-checked by the first reviewer (ASC) and checked and validated by the second and third reviewers (ARP and JVC).

Text coding and the construction of descriptive themes were performed by identifying the main topics presented in the descriptions of results, discussion, and conclusions of the included studies and aggregating similarities between studies. Thereafter, the descriptive themes were grouped into 2 main analytical themes—facilitators of telemedicine and barriers to telemedicine use—which were then subdivided into smaller topics.

The characterization of the main challenges and opportunities posed by the adoption of telemedicine services was performed by interpreting the results of the thematic synthesis.

## Results

### Included Studies

**Figure 1:**
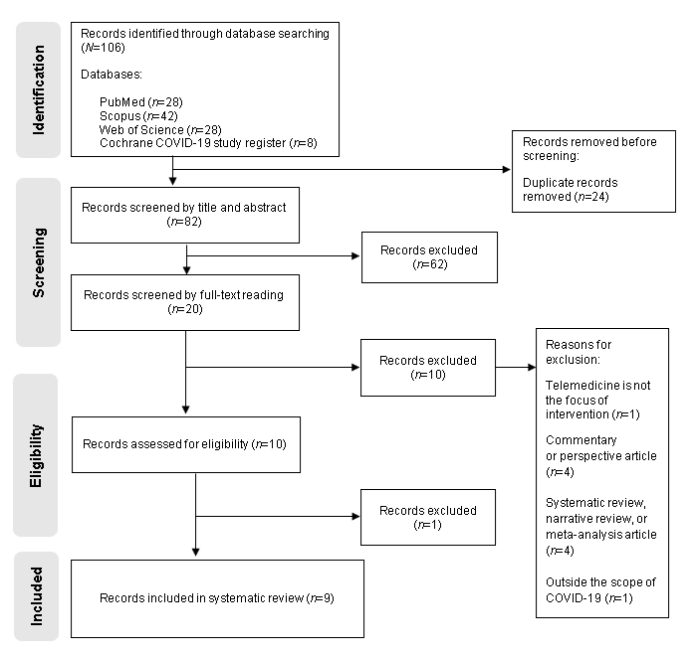
PRISMA (Preferred Reporting Items for Systematic Reviews and Meta-Analyses; 2020) flowchart.

We obtained 106 references resulting from the literature search: 28 from PubMed, 42 from Scopus, 28 from Web of Science, and 8 from the Cochrane COVID-19 Study Register (Figure 1). Of these, 24 duplicate references were removed and 82 advanced to title and abstract analysis. Therefore, 62 (N=106, 58.5%) articles were excluded for not meeting the eligibility criteria, and 20 (N=106, 18.9%) advanced to full reading analysis. At this stage, 10 (N=106, 9.4%) articles were excluded for not meeting the eligibility criteria and 10 (N=106, 9.4%) articles were subject to quality assessment (Figure 1). Quality assessment resulted in the rejection of 1 of the selected articles [57] because of insufficient methodological robustness (Multimedia Appendix 2). Although this review initially aimed to search for qualitative and quantitative evidence, the 9 articles selected presented a descriptive quantitative approach. Therefore, all selected articles were evaluated under the quantitative descriptive studies category of the quality assessment of the included articles based on the Mixed Methods Appraisal Tool (Multimedia Appendix 2).

In this review, we selected 9 (N=106, 8.5%) articles [58-66]. Figure 1 illustrates the selection process, including the reasons for article exclusion, according to the PRISMA flowchart [43].

### Study Characteristics

Regarding publication date, 2 (N=9, 22%) articles were published in 2020 [64,66], 5 (N=9, 56%) articles in 2021 [58-60,63,65], and 2 (N=9, 22%) articles in 2022 [61,62]. All articles were published in the English language and were conducted in the United States, comprising 3 (N=9, 33%) cross-sectional studies [58,59,66], 1 (N=9, 11%) cohort study [60], 3 (N=9, 33%) retrospective cohort studies [62-64], 1 (N=9, 11%) cross-sectional study with comparative analysis [61], and 1 (N=9, 11%) mixed methods study [65] (Multimedia Appendix 3).

### Facilitating Factors in the Use of Telemedicine Services

We identified 6 factors that facilitated the use of telemedicine services, which were mentioned in 8 (89%) of the 9 reviewed studies [58-64,66] (Table 1).

The level of health insurance coverage was identified as a facilitating factor for the use of telemedicine in 6 reviewed studies. In the study conducted by Chen et al [62], people with Medicare coverage were more likely to have telemedicine consultations via telephone or video than those who used their private health insurance or those who had no insurance associated [62]. Other studies included in this systematic review [60,61,63,64,66] also found that patients with insurance coverage for telemedicine services, or those who accessed these services using their private health insurance (vs employer or state health insurance), were more likely to have an internet-based consultation (Table 1).

**Table 1:** List of barriers to the use of telemedicine services to access medical specialty consultations during the COVID-19 pandemic (N=9).

Facilitating factors	Studies where facilitating factors were addressed or mentioned	Total, n (%)
Health insurance coverage	[60-64,66]	6 (67)
Prevention of SARS-CoV-2 infection	[58,59,62]	3 (33)
Access to internet services	[59,60,62]	3 (33)
Access to technological devices	[59,60,62]	3 (33)
Better management of work-life balance	[58]	1 (11)
Travel cost savings	[58]	1 (11)

The prevention of SARS-CoV-2 infection was also identified as a facilitating factor for telemedicine use. Albon et al [58] and Chen et al [62] identified that one of the reasons why patients used telemedicine services was to avoid personal contacts that could potentially lead to illness from COVID-19. This factor was more important for patients with regular follow-up appointments [58,62]. In addition, Ng and Park [59] also observed that the presence of patient comorbidities was associated with greater accessibility to telemedicine services as a replacement for regularly scheduled appointments to prevent and reduce the risk of contracting COVID-19 (Table 1).

Lattimore et al [60], Elam et al [61], and Ng and Park [59] highlighted the relevance of access to internet services and technological devices as key factors in accessing telemedicine services. People who lived in areas with faster internet services and those who had technological devices at their disposal, such as smartphones, tablets, or computers, were more likely to perform and choose telemedicine consultations [59-61] (Table 1).

The reviewed studies also identified management of work-life balance and savings on travel costs as facilitating factors for telemedicine use. According to Albon et al [58], about half of the care programs for people with cystic fibrosis (49%) identified reduced travel costs and easier management of family and professional life as the main factors facilitating the use of telemedicine compared with face-to-face consultations (Table 1).

### Barriers to the Use of Telemedicine Services

The lack of necessary technological conditions or equipment and the lack of home internet service, including broadband internet service, were identified as barriers to telemedicine specialty consultations during the COVID-19 pandemic in 8 reviewed studies [58-65] (Table 2). Regarding the lack of technological conditions or equipment needed, the authors specifically highlighted the lack of access to smartphones and the fact that some people do not have devices with an incorporated camera [58-65].

**Table 2:** List of barriers to the use of telemedicine services to access medical specialty consultations during the COVID-19 pandemic (N=9).

Barriers	Studies where barriers were addressed or mentioned	Total, n (%)
Lack of access to internet services	[58-65]	8 (89)
Lack of access to technological devices	[58-65]	8 (89)
Racial and ethnic disparities	[58,59,61,62,64,66]	6 (67)
Low level of digital literacy	[60-62,64-66]	6 (67)
Low income	[58-60,63,64,66]	6 (67)
Age (years)	[59,61-64]	5 (56)
Language barriers	[58,62,64,65]	4 (44)
Health insurance coverage	[63,64]	2 (22)
Data privacy and confidentiality concerns	[64]	1 (11)
Geographic disparities (area of residence)	[59]	1 (11)
Need to perform complementary diagnostic tests or receive test results	[65]	1 (11)

Several reviewed studies [58,59,61,62,64,66] identified belonging to racial and ethnic minority groups as an obstacle to accessing telemedicine consultations (Table 2). Eberly et al [64] mentioned that people belonging to racial and ethnic minority groups generally have more difficulties accessing health care, have developed weaker relationships with health care professionals, and have reported more negative experiences in health care in the past. These factors were identified by the authors as leading to more difficulties in accessing telemedicine consultations or leading to less referrals [64]. Similarly, Albon et al [58] reported that people from racial and ethnic minority groups were less referred to telemedicine consultations, had more concerns about associated copayments, and presented language barriers.

A lower level of digital literacy was also identified in reviewed studies as an obstacle for telemedicine consultations during the COVID-19 pandemic [61,62,64-66] (Table 2). Furthermore, this obstacle has been associated with other circumstances. Lattimore et al [60] and Chen et al [62] associated low digital literacy with lower use of telemedicine services, especially among older people. Lattimore et al [60] also associated low literacy with a lower socioeconomic level and less access to technological resources. Nonetheless, Haynes et al [65] reported that digital literacy can be low even in groups with access to technology.

Some reviewed studies also identified low income as an obstacle to telemedicine consultation access during the COVID-19 pandemic [58-60,63,64,66] (Table 2). Eberly et al [64], Darrat et al [63], and Lattimore et al [60] showed that people with lower household incomes were less likely to access internet services and technologies and were less associated with telemedicine consultations [60,63,64]. Ng and Park [59] reported that disparities in accessibility to telemedicine services by income level were also observed. Those with lower incomes were referred less frequently to telemedicine consultations by health professionals [59]. In the study by Albon et al [58], people who reported experiencing financial problems during the study period found it more difficult to use telemedicine services. Whaley et al [66] also observed that people who lived in locations associated with lower income levels experienced smaller reductions in in-person visits while also experiencing lower rates of telemedicine adoption than people living in higher-income zip codes.

Age is highlighted in 5 reviewed articles as a barrier to the adoption of telemedicine medical specialty consultations during the COVID-19 pandemic [59,61-64] (Table 2). In particular, patients aged >55 years had fewer telemedicine consultations [59,61,62,64]. Eberly et al [64] indicated that older users feel more concerned about privacy violations and the insecurity of their personal data. This factor led to more resistance to telemedicine consultations (Table 2).

The existence of language barriers was also recognized as a limitation in accessing telemedicine specialty consultations during the COVID-19 pandemic [58,62,64,65] (Table 2). The studies conducted by Albon et al [58], Haynes et al [65], and Eberly et al [64] revealed that people who were not native language speakers were less likely to undergo an internet-based appointment and were more likely to undergo face-to-face consultations. Chen et al [62] also reported similar findings.

In coherence with the identification of health insurance coverage as a facilitating factor, the lack of such coverage was identified as a barrier to accessing telemedicine medical consultations during the COVID-19 pandemic [63,64] (Table 2). Eberly et al [64] found that Medicaid insurance beneficiaries used fewer telemedicine consultations than private health insurance users and discussed the need to have parameterization in telemedicine consultation payments between insurers through permanent legislative action.

In the study by Ng and Park [59], disparities in access to telemedicine specialty consultations during the COVID-19 pandemic were found according to area of residence. People who lived outside metropolitan areas reported being referred less often for telemedicine consultations by health professionals [59] (Table 2).

Other obstacles to accessing telemedicine specialty consultations during the COVID-19 pandemic were also found. In particular, the need to perform complementary diagnostic tests or to deliver medical test results was identified by Haynes et al [65] (Table 2). This study specifically mentioned the need to undergo laboratory tests or personally receive clinical records of insulin levels as reasons why some people opted for in-person appointments as an alternative to telemedicine [65].

**Figure 2:**
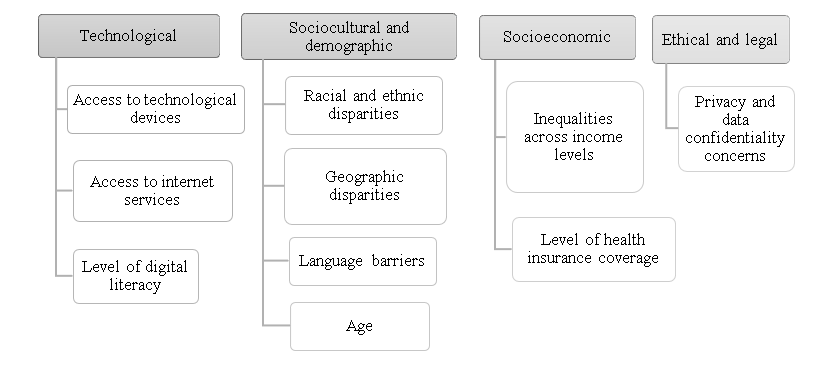
Opportunities and challenges to the adoption of telemedicine services to access medical specialty consultations during the COVID-19 pandemic.

Overall, results from the reviewed studies showed that opportunities and challenges to access telemedicine specialty consultations during the COVID-19 pandemic are broad and multifactorial (Figure 2).

## Discussion

### Interpretation of Results

Thematic synthesis of the reviewed studies identified more barriers to the use of telemedicine than facilitating factors (11 barriers and 6 facilitating factors). This finding may suggest that the existing conditions for the use of telemedicine before the COVID-19 pandemic were not ready to respond to the rapid transition to a new reality of medical care delivery based on physical distancing. This transition might, in turn, have worsened preexisting disparities in health care delivery [25,32,33].

Management of work-life balance, saving travel costs, and prevention of SARS-CoV-2 infection were the factors that, according to reviewed evidence, most encouraged the use of telemedicine for specialty consultations during the pandemic period [58,59,62]. Remote medical appointments can be more comfortable for patients by allowing them to avoid traveling to the appointment locations and reducing the associated costs [16,32,67]. Telemedicine can also adapt medical appointment timings to personal and professional routines and provide more flexibility [16,32,67]. In addition, and given the context under analysis, the reduction of in-person consultations as a COVID-19 prevention measure has forced people to access health care by alternative means, and telemedicine is one of the most appropriate digital solutions for that purpose [27,68,69]. These 2 results are consistent with the systematic review conducted by Almathami et al [30], which also identified time savings, patient convenience, management, accessibility, and cost reductions as facilitating factors for making home health care appointments web based [30]. Furthermore, telemedicine consultations can proceed synchronously (in real time or through video consultation) or asynchronously (through the transmission of data and information indirectly, via digital platforms), providing individual advantages not only in terms of time, costs, and travel savings but also in terms of reducing costs and expenses for health systems [17,27].

This review also showed that patients whose insurance had coverage for telemedicine services, accessed more virtual consultations [60-64,66]. Conversely, people with more concerns about associated copayments, users of public health insurance, and those whose insurance did not cover telemedicine services accessed this type of consultation less [63,64].

These findings are closely related to inequalities in access to telemedicine across income levels, which we also identified to be a barrier to telemedicine specialty consultations during the COVID-19 pandemic [58-60,63,64,66]. These findings are in line with results from other studies [15,17,70-75]. In the narrative review by Shaw et al [70], reduced telemedicine access was observed in lower-income communities. Furthermore, Barbosa et al [17] and Adigwu et al [71] found that patients from lower-income areas also had less access to medical specialty consultations during the pandemic period. Moreover, Lesher et al [72] found that lower income levels were associated with lower use of internet-based surgical consultations.

Regarding technological factors, according to reviewed studies, expanded access to internet services and technological devices can increase the chance of accessing telemedicine consultations [59-61]. Conversely, not having access to digital devices or the internet can limit access to telemedicine services or even make it impossible [58-65]. The lack of access to internet and broadband services, poor signal coverage, a lack of technological devices suitable for conducting internet-based consultations, the absence of built-in cameras, or devices with poor video and audio transmission are some of the barriers to the use of telemedicine also identified by Almathami et al [30] and Shaw et al [70]. Other studies have also identified technological barriers as a major problem for telemedicine implementation during the pandemic period [2,32,76-78].

In parallel, low levels of digital literacy imply reduced access to telemedicine consultations because the lack of familiarization and knowledge about technology is strongly related to mistrust and fear, leading to resistance [61,62,64-66]. Digital literacy is a widely studied topic and is extremely important for the success of the digital transition in all areas of society [2,79-84]. Globally, there are several initiatives and recommendations generated to promote digital health literacy education, mainly targeting the most susceptible populations [85].

Regarding sociocultural and demographic challenges, this systematic review identified 1 study that noted geographic disparities in referrals to telemedicine consultations [59]. In this study, people living in metropolitan areas were more often referred to telemedicine consultations than people living in nonmetropolitan or rural areas [59]. Although telemedicine was originally designed to respond to the challenges of health care access in remote locations, people living in rural areas are more likely to have lower income levels, less access to broadband internet services and technological infrastructure, and potentially lower levels of digital literacy, which constitute barriers to accessing digital care [28,31,35,80,86]. In parallel, according to Ng and Park [59], the low referral rate to telemedicine consultations in rural areas during the pandemic may also be related to the fact that, in these places, COVID-19 containment measures were implemented later and were less restrictive.

Disparities among people belonging to racial and ethnic minority groups were also identified as a barrier to telemedicine access during the COVID-19 pandemic. Six studies included in this review [58,59,61,62,64,66] identified that patients belonging to minority groups were the most fragile in terms of access to telemedicine. This observation is not surprising, as these patients have been previously shown to face more difficulties in accessing medical care in general. Furthermore, it illustrates that besides being an effective tool to minimize disparities, telemedicine adoption can also deepen existing inequalities [35,86-89]. The main reasons for racial and ethnic disparities in access to telemedicine consultations during the COVID-19 pandemic were the lack of technological resources, less familiarity with these technologies, less referral by health professionals, fear of the possible associated copayments, and the existence of language barriers [32,70,90].

Importantly, the existence of language limitations was also identified in 4 studies in this review [58,62,64,65] as a barrier to the use of telemedicine during the COVID-19 pandemic. People with less proficiency in the language of the country they lived in recurred more to face-to-face consultations [64]. Other studies have identified this barrier and reinforced the importance of including translation services in all stages of the health care interaction to increase adherence to health guidance [25,76,79,90-92]. The study conducted by Tatemoto et al [93] identified communication barriers as the main obstacle to telerehabilitation therapy, emphasizing the importance of education for the use of telemedicine, along with language translation during the interaction. Another inclusive measure could be to provide educational training for the use of telemedicine in several languages [94].

This review also identified that different studies described that patients aged >55 years had fewer medical specialty consultations via telemedicine during the COVID-19 pandemic [59,61-64]. This age-related barrier has been shown to be linked with low digital literacy levels, which leads to reluctance in technological adoption [80,95-97]. Furthermore, older patients tend to accumulate more comorbidities and general physical limitations, which complicate remote medical consultations, such as a difficulty to see or hear and poor manual dexterity [32,98]. These limiting factors often lead to frustration and demotivation to use technology, requiring the assistance of caregivers [99,100]. However, the higher disease burdens in older patients translate into the need for more medical consultations. Therefore, overcoming age-associated barriers to telemedicine adoption is fundamental so that older patients benefit from remote medical appointments, thereby reducing travel costs and risks while providing more comfort [101].

Performing complementary diagnostic tests and delivering medical results were also factors that, according to Haynes et al [65], have led patients to opt for in-person consultations. Nevertheless, the execution of laboratory tests or other complementary tests should not be automatically considered a barrier to the use of telemedicine solely based on the assumption that they require in-person attendance [102-104]. An increasing number of medical devices (hardware) remotely register and collect vital and anthropometric signs (pulse, glycemia, temperature, body fat, etc) and perform diagnostic tests, such as an electrocardiogram [13,105-108]. Furthermore, the delivery of medical records can already be done by the patient through dedicated digital platforms (software) [109]. However, digital health integration does not work per se and should consider the limitations related to technology access and use, as previously discussed [13,110].

Data privacy and confidentiality concerns have also been identified as an ethical and legal challenge to telemedicine specialty consultations during the COVID-19 pandemic [64]. Results suggest that certain patients may feel insecure about scheduling medical appointments using digital platforms, as they have concerns about their privacy [111-115]. Health data are sensitive, confidential, and private [116]. To provide medical care, health data must be accessed by different professionals and sometimes institutions and across different devices [117]. As accumulating evidence suggests that health digital data systems are vulnerable and outdated, the treatment of health data in digital spaces can sometimes pose a higher risk of breaches and damages [118-122]. Telemedicine consultations must preserve the patient-physician relationship, which requires the implementation of data protection safeguards and respect for medical confidentiality [123-127]. Therefore, it is crucial to continue implementing strategies at institutional, national, and international levels that promote the responsible use of medical data, including the adoption of telemedicine-specific safeguards [119,125-127]. These might involve implementing codes of conduct, optimizing ethical rules, establishing fair procedures, and imposing dissuasive sanctions (disciplinary, legal, and social) for misconduct [119].

### Strengths and Limitations

Similar to any other study, this review is not completely free of limitations. First, all the studies that were included in this review originate from the United States. Therefore, it is important to exercise caution when extrapolating the conclusions to different contexts. Furthermore, only articles written in English, Spanish, and Portuguese were searched, which could be a potential limitation as relevant results from other studies written in different languages may have been excluded from this systematic review. Most of the included studies are retrospective and used large-volume databases to extract relevant information. The possibility of errors in coding during data input or failures in the analysis and interpretation of statistical associations could not be excluded. Moreover, another aspect that should be considered is that this review did not include any “gray literature,” which could have limited access to contrasting or unknown information. In addition, the collection of primary studies only involved searches using MeSH terms, which could have impacted the number of articles identified through our search. In parallel, the fact that this systematic review relied on a synthesis of qualitative evidence may also have been subject to possible interpretation biases. In this review, an objective assessment of study bias was made in the selection process. However, the results are not completely free of human judgment.

Nonetheless, this systematic review adds a body of evidence-based knowledge about the facilitating factors and barriers to telemedicine use for hospital medical specialty consultations in the context of the COVID-19 pandemic. The protocol of our review was also previously submitted and published in PROSPERO to ensure transparency [44]. This review searched 4 well-known databases using key terms registered in MeSH and included only indexed and peer-reviewed articles. A total of 3 reviewers were involved in this study throughout the execution process. Article selection was performed blindly and independently, and study quality was thoroughly assessed. All included studies involved large sample numbers, which is indicative of scientific robustness.

### Implications of the Results for Clinical Practice, Policy, and Future Research

By examining the facilitating factors of and barriers to telemedicine adoption during a time when in-person health care was limited by the existence of a public health emergency, the results of this systematic review highlight the need to continue rethinking how distance health care is delivered.

Several challenges need to be overcome to take advantage of the full potential of digital technology in health care. In particular, this review highlights the importance of paying special attention to the needs of the most susceptible and underprivileged individuals in digital care. It also underscores the necessity of ensuring fair and equitable access to technological resources, providing adequate incentives for medical referrals, and strengthening public health policies that promote digital health literacy. In addition, at the clinical level, it is crucial to establish standards that dictate the appropriate use of telemedicine services, accredit professionals and institutions to provide these services, reinforce administrative regulations, and establish robust legal frameworks that safeguard users and institutions against potential breaches in data system security.

Future research should examine whether the identified opportunities and challenges in accessing telemedicine services during the pandemic period in the United States apply to other international contexts. In addition, research should explore how these opportunities and challenges apply to nonpandemic settings and different populations, as well as strategies to optimize the benefits and minimize the risks of using telemedicine for medical specialty consultations across various settings.

### Conclusions

In the last few years, telemedicine adoption has been increasing. More recently, telemedicine has emerged as a powerful ally for safe and effective health care delivery during a public health emergency. However, the COVID-19 pandemic exacerbated disparities in telemedicine access.

We found that health insurance coverage was a primary factor that facilitated the use of telemedicine services, although limited access to technology and internet services were the most significant barriers to adherence to this mode of care. To expand health care access and provide high-quality care for all, including the most susceptible communities, the determinants of the technological, sociocultural, demographic, socioeconomic, ethical, and legal challenges identified in this systematic review must be researched and met with informed and dedicated responses. These results underscore the importance of rethinking how remote health care is delivered to ensure fair and equitable access, especially during a public health crisis when digital health care is crucial.

## Appendix

Multimedia Appendix 1 PRISMA (Preferred Reporting Items for Systematic Reviews and Meta-Analyses; 2020) checklist.

Multimedia Appendix 2 Results of the quality assessment of the included articles based on the Mixed Methods Appraisal Tool (version 2018).

Multimedia Appendix 3 Data extraction table: characterization of the included studies.

## Abbreviations

MeSH: Medical Subject Headings
PICOS: Population, Intervention, Comparison, Outcome, and Study Design
PRISMA: Preferred Reporting Items for Systematic Reviews and Meta-Analyses
WHO: World Health Organization
