# Supplementary material for: Facilitators of and Barriers to Accessing Hospital Medical Specialty Telemedicine Consultations During the COVID-19 Pandemic: Systematic Review
Source: J Med Internet Res. 2023 Jul 10;25:e44188. doi: 10.2196/44188 (PMC10337395; doi:10.2196/44188)
Supplement: Multimedia Appendix 2 [file jmir_v25i1e44188_app2.docx]

| Authors | Category of study design | Methodological quality criteria | Yes | No | Can´t tell |
| --- | --- | --- | --- | --- | --- |
| Albon et al., 2021 | Screening questions | S1. Are there clear research questions? | X |  |  |
|  |  | S2. Do the collected data allow to address the research questions? | X |  |  |
|  | 4.Quantitative descriptive | 4.1. Is the sampling strategy relevant to address the research question? | X |  |  |
|  |  | 4.2. Is the sample representative of the target population? |  |  | X |
|  |  | 4.3. Are the measurements appropriate? | X |  |  |
|  |  | 4.4. Is the risk of nonresponse bias low? |  |  | X |
|  |  | 4.5. Is the statistical analysis appropriate to answer the research question? | X |  |  |
| Lattimore et al., 2021 | Screening questions | S1. Are there clear research questions? | X |  |  |
|  |  | S2. Do the collected data allow to address the research questions? | X |  |  |
|  | 4.Quantitative descriptive | 4.1. Is the sampling strategy relevant to address the research question? |  |  | X |
|  |  | 4.2. Is the sample representative of the target population? |  |  | X |
|  |  | 4.3. Are the measurements appropriate? | X |  |  |
|  |  | 4.4. Is the risk of nonresponse bias low? | X |  |  |
|  |  | 4.5. Is the statistical analysis appropriate to answer the research question? | X |  |  |
| Elam et al., 2022 | Screening questions | S1. Are there clear research questions? | X |  |  |
|  |  | S2. Do the collected data allow to address the research questions? | X |  |  |
|  | 4.Quantitative descriptive | 4.1. Is the sampling strategy relevant to address the research question? | X |  |  |
|  |  | 4.2. Is the sample representative of the target population? |  |  | X |
|  |  | 4.3. Are the measurements appropriate? | X |  |  |
|  |  | 4.4. Is the risk of nonresponse bias low? | X |  |  |
|  |  | 4.5. Is the statistical analysis appropriate to answer the research question? | X |  |  |
| Chen, Andoh e Nwanyanwu, 2022 | Screening questions | S1. Are there clear research questions? | X |  |  |
|  |  | S2. Do the collected data allow to address the research questions? | X |  |  |
|  | 4.Quantitative descriptive | 4.1. Is the sampling strategy relevant to address the research question? | X |  |  |
|  |  | 4.2. Is the sample representative of the target population? |  |  | X |
|  |  | 4.3. Are the measurements appropriate? | X |  |  |
|  |  | 4.4. Is the risk of nonresponse bias low? | X |  |  |
|  |  | 4.5. Is the statistical analysis appropriate to answer the research question? | X |  |  |
| Ng e Park, 2021 | Screening questions | S1. Are there clear research questions? | X |  |  |
|  |  | S2. Do the collected data allow to address the research questions? | X |  |  |
|  | 4.Quantitative descriptive | 4.1. Is the sampling strategy relevant to address the research question? | X |  |  |
|  |  | 4.2. Is the sample representative of the target population? |  |  | X |
|  |  | 4.3. Are the measurements appropriate? | X |  |  |
|  |  | 4.4. Is the risk of nonresponse bias low? | X |  |  |
|  |  | 4.5. Is the statistical analysis appropriate to answer the research question? | X |  |  |
| Darrat et al., 2021 | Screening questions | S1. Are there clear research questions? | X |  |  |
|  |  | S2. Do the collected data allow to address the research questions? | X |  |  |
|  | 4.Quantitative descriptive | 4.1. Is the sampling strategy relevant to address the research question? | X |  |  |
|  |  | 4.2. Is the sample representative of the target population? |  |  | X |
|  |  | 4.3. Are the measurements appropriate? | X |  |  |
|  |  | 4.4. Is the risk of nonresponse bias low? | X |  |  |
|  |  | 4.5. Is the statistical analysis appropriate to answer the research question? | X |  |  |
| Eberly et al., 2020 | Screening questions | S1. Are there clear research questions? | X |  |  |
|  |  | S2. Do the collected data allow to address the research questions? | X |  |  |
|  | 4.Quantitative descriptive | 4.1. Is the sampling strategy relevant to address the research question? | X |  |  |
|  |  | 4.2. Is the sample representative of the target population? |  |  | X |
|  |  | 4.3. Are the measurements appropriate? | X |  |  |
|  |  | 4.4. Is the risk of nonresponse bias low? | X |  |  |
|  |  | 4.5. Is the statistical analysis appropriate to answer the research question? | X |  |  |
| Haynes et al., 2021 | Screening questions | S1. Are there clear research questions? | X |  |  |
|  |  | S2. Do the collected data allow to address the research questions? | X |  |  |
|  | 4.Quantitative descriptive | 4.1. Is the sampling strategy relevant to address the research question? | X |  |  |
|  |  | 4.2. Is the sample representative of the target population? |  |  | X |
|  |  | 4.3. Are the measurements appropriate? | X |  |  |
|  |  | 4.4. Is the risk of nonresponse bias low? | X |  |  |
|  |  | 4.5. Is the statistical analysis appropriate to answer the research question? | X |  |  |
| Barry et al., 2020 | Screening questions | S1. Are there clear research questions? |  | X |  |
|  |  | S2. Do the collected data allow to address the research questions? |  |  | X |
| Whaley et al., 2020 | Screening questions | S1. Are there clear research questions? | X |  |  |
|  |  | S2. Do the collected data allow to address the research questions? | X |  |  |
|  | 4.Quantitative descriptive | 4.1. Is the sampling strategy relevant to address the research question? |  |  | X |
|  |  | 4.2. Is the sample representative of the target population? | X |  |  |
|  |  | 4.3. Are the measurements appropriate? | X |  |  |
|  |  | 4.4. Is the risk of nonresponse bias low? | X |  |  |
|  |  | 4.5. Is the statistical analysis appropriate to answer the research question? | X |  |  |
