# Supplementary material for: Facilitators of and Barriers to Accessing Hospital Medical Specialty Telemedicine Consultations During the COVID-19 Pandemic: Systematic Review
Source: J Med Internet Res. 2023 Jul 10;25:e44188. doi: 10.2196/44188 (PMC10337395; doi:10.2196/44188)
Supplement: Multimedia Appendix 3 [file jmir_v25i1e44188_app3.docx]

| Authors, date and country of publication | Study design | Period of data collection | Sample, setting and medical specialty/type of care | Objectives | Description of the intervention | Main results |
| --- | --- | --- | --- | --- | --- | --- |
| Albon et al., 2021  EUA | Cross-sectional study | 24 August to 30 October 2020.  29 July and 18 September 2020 | N= 424+286 programs.  Medical specialty: Pulmonology  (Cystic Fibrosis - CF). | Understanding perceptions and use of telehealth; and identifying facilitators and barriers to the use of telehealth, among people with CF and their families, with diverse socio-economic and ethnic backgrounds. | Descriptive statistical analysis of the 2020 QF healthcare status surveys completed by (1) people with CF, by (2) CF Healthcare Program Directors and by (3) the CF Foundation User Register. | (1) People from racial and ethnic minorities and people who reported financial problems had less access to telemedicine consultations.  (2) The elimination of insurance company copayments increased coverage and access to telemedicine services. |
| Lattimore et al., 2021  EUA | Cohort study | 1 July 2019 to 31 May 2021 | N= 14792 patients in the surgical department of a university tertiary care Centre (Virginia).  Medical specialties:  Surgery (General, Breast, Colorectal, Cardiothoracic, Bariatric, Oncological, Paediatric, Transplant and Vascular). | To examine demographic and socioeconomic differences in the use of telemedicine in surgical patients during the pandemic of COVID-19. | Patients from the surgical department were divided into three types of visits: pre-COVID-19 face-to-face, COVID-19 face-to-face, or COVID-19 telemedicine. Encounter data were extracted from institutional databases. Generalized linear models were used to examine associations of gender, race/ethnicity, Distressed Communities Index score, activation of MyChart (a digital portal for users to access their medical record and communicate with clinicians), and insurance status with telemedicine use during the COVID-19 pandemic. | (1) There were no significant differences in access to telemedicine consultations between races/ethnicities.  (2) In the first three months of the COVID-19 pandemic, telemedicine was used more by women and by people without government or commercial insurance.  (3) Telemedicine visits during COVID- 19 were more likely in people from less socioeconomically distressed cities, who used a MyChart, and with non-government or commercial insurance. |
| Elam et al., 2022  EUA | Cross-sectional study with comparative analysis | 30 April to 25 May 2020 | N= 1720 patients who had a scheduled visit at the Kellogg Eye Center at the University of Michigan.  Medical specialty: Ophthalmology | To evaluate the relationship between telemedicine use and socio-demographic factors of users who sought ophthalmic care. | Review of eye care utilization patterns of a stratified random sample of 1720 patients and calculated their odds of having a video, telephone or in-person visit compared with a rescheduled visit using a multinomial logistic regression model. | (1) 31.2% of people had face-to-face consultations; 18.6% had consultations by telephone; 5.5% had video consultations; and 44.7% had rescheduled consultations. (2) The study population was mostly white (80.2%).  (3) Black (and other) people were less likely to have in-person appointments.  (4) People living further away from the hospital Centre were more likely to have video, telephone or in-person appointments than rescheduled appointments.  (5) People with access to faster internet service were less likely to have a face-to-face consultation.  (6) Older people were more likely to have an in-person appointment and less likely to have video or telephone call appointments.  (7) People of non-white race had less access to health care. |
| Chen, Andoh e Nwanyanwu, 2022  EUA | Retrospective cohort study | 1 March to 31 August 2020 | N= 5023 patients of an ophthalmic academic centre (Yale New Haven Hospital health system).  Medical specialty: Ophthalmology. | Identify disparities in the use of telemedicine during the COVID-19 pandemic. | Collection of demographic information through electronic medical records and consequent identification of socioeconomic characteristics by zip code-level, which were drawn from the 2019 American Community Survey 5-year estimates. | (1) A total of 8116 clinical encounters were completed for 5023 service patients. 8.9% participated in a video encounter, 12.8% completed a telephone encounter, and 78.3% attended clinical appointments in person.  (2) Ethnic/racial minorities, older people, and non-English speakers were significantly less likely to complete a telemedicine encounter.  (3) Among users who completed any type of telemedicine appointment, the characteristics older age, Medicare insurance, and black race were associated with making only telephone consultations. |
| Ng e Park, 2021  EUA | Estudo transversal | 10 June 2020 to 15 July 2020 | N= 6172 MEDICARE beneficiaries aged 65 and over.  Medical specialty:  not specified. | To examine the factors associated with accessibility of telehealth services during the COVID-19 pandemic among adults aged 65 years and over. | Two weighted multivariable logistic regression models were used to examine associations between usual health care providers offering telehealth appointments:  (1) during the COVID-19 pandemic and (2) to replace a regularly scheduled appointment.  Factors including sociodemographic characteristics, comorbidities, digital access, and literacy were examined. | (1) 81.2% of respondents reported that their usual healthcare providers offered telehealth consultations during the COVID-19 pandemic.  (2) 56.8% reported that their usual providers offered telehealth services to reschedule a regularly scheduled appointment.  (3) Disparities in accessibility of telehealth services were observed by gender, area of residence, income level, and US Census tract region.  (4) Beneficiaries with Internet access who reported ever participating in video, voice, or Internet conferencing calls before were more likely to report having access to telehealth.  (5) Non-Hispanic black beneficiaries and beneficiaries with comorbidities were more likely to have their usual provider offer telehealth to replace a regularly scheduled appointment. |
| Darrat et al., 2021  EUA | Retrospective cohort study | 17 March to 1 May 2020 | N= 1162 patients of the Otorhinolaryngology Department - Head and Neck Surgery, of a tertiary academic centre;  Medical specialty: Otorhinolaryngology. | To assess the demographic and socioeconomic factors associated with patients access to telehealth services during the COVID-19 pandemic. | Univariate and multivariate logistic regression models were created to compare (1) completion of a virtual visit compared with another type of visit to identify demographic and socioeconomic factors associated with the ability to adopt telehealth; (2) completion of a virtual visit compared with a telephone visit to identify individuals without time-sensitive issues who were unable to access virtual care but were able to access care through other means; and (3) completion of virtual or telephone visits compared with non-face-to-face visits. | (1) Of the 1162 clients included, 990 visits were made and 437 of these completed a virtual consultation.  (2) Female and insured users were more likely to engage in virtual consultations.  (3) Age, gender, average household income and marital status are associated with patient participation in telehealth.  (4) Vulnerable patient populations are less likely to access telehealth consultations.  (5) Users in areas with lower median household incomes, who had Medicaid or were uninsured, and who were older were more likely to engage in telehealth consultations. |
| Eberly et al., 2020  EUA | Retrospective cohort study | 16 March to 11 May 2020 | N= 148402 patients who booked a telemedicine consultation in a university health system.  Medical Specialties: Cardiology, pneumology, rheumatology, gastroenterology, infectious diseases, rheumatology, nephrology, haematology-oncology, and general and family medicine. | To assess whether inequities exist between clients who completed a telemedicine encounter (by telephone or video) with those who were scheduled but did not complete a telemedicine visit during the pandemic of COVID-19. | Application of multivariate models to assess the association between sociodemographic factors, including gender, race/ethnicity, socioeconomic status and language, and the use of telemedicine visits, as well as specific video use. | (1) Of 148 402 patients who had scheduled telemedicine visits during the study period, only 80780 users (54.4%) completed visits.  (2) Of 78539 patients with completed visits, 45.6% were conducted via video, while 54.4% conducted telephone visits.  (3) The variables: advanced age, Asian race, non-English as an official language, and Medicaid insurance were independently associated with fewer completed telemedicine visits.  (4) The variables advanced age, female gender, black race, Latino ethnicity, and lower household incomes were associated with less use of video for telemedicine visits.  (5) Middle incomes were associated with greater use of telemedicine services. However, the same was not observed in middle-high incomes. |
| Haynes et al., 2021  EUA | Cross-sectional study and retrospective cohort study | 19 March to 30 June 2020 | N= 1292 patients of the UC Davis health unit with Diabetes Mellitus (DM) type I or II.  Medical specialty: Endocrinology. | To identify factors associated with users regarding the uptake of telemedicine services for the clinical subspecialty of DM during the COVID-19 pandemic. | Use of multivariate logistic regression to explore the associations between telemedicine use and demographic factors; and subsequently surveyed users who received face-to-face care to understand the reasons for non-use of telemedicine services. | (1) During the analyzed period, from the 1292 users seen, 552 received at least one telemedicine consultation and 740 preferred face-to-face or telephone consultations.  (2) A lower association was found between people aged over 65 years and use of telemedicine services.  (3) People who do not have English as a primary language or who need translation services subscribed less to telemedicine services.  (4) People with public insurance used telemedicine services less than those who used private insurance.  (5) People who preferred face-to-face consultations believe that telemedicine consultations have less quality, that they are not familiar with the technology and accused having less access to smartphones.  (6) Most people reported not wanting to use or seek telemedicine services for diabetes in the future. |
| Whaley et al., 2020  EUA | Cross-sectional study | March to April 2018, 2019 and 2020 | N = 6.8 million people from the 50 United States (US) states who received health insurance through their employers.  Type of care:  Preventive care (colonoscopy, mammogram, hemoglobin levels, and vaccination); Elective care (musculoskeletal surgery, cataract surgery, MRI); Non-elective care (Angioplasty, chemotherapy, maternity care); Prescription drugs (Statin; antidiabetics; Antiasthmatics) | To examine changes in health care utilization that occurred during March and abri 2020 relative to the same period for 2019 and 2018 in patients of employer-provided insurance.  Examine whether changes differed by zip code-level, race/ethnicity, or income. | Evaluation of the trend in utilization of preventive services, non-group care, elective procedures, prescription drugs, in-person office visits, and telemedicine visits during the first 2 months of the 2020 COVID-19 pandemic compared to existing trends in 2019 and 2018.  Disparities in pandemic association with healthcare utilization based on race and patient income at the zip code-level were also analyzed. | (1) The study had equal representation of people by age, gender, risk score and geographic distribution over the three years analyzed.  (2) A large portion of the US population with employer insurance, in the first 2 months of pandemic COVID-19, used less preventive and elective care.  (3) Telemedicine use increased rapidly, but not enough to account for reductions in in-person primary care visits.  (4) Patients living in postcodes with lower-income or racial/ethnic minority populations, experienced smaller reductions in in-person visits, but also had lower rates of telemedicine adoption. |
